# Generation and evaluation of a monoclonal antibody, designated MAdL, as a new specific marker for adenocarcinomas of the lung

**DOI:** 10.1038/bjc.2011.281

**Published:** 2011-08-02

**Authors:** H Schultz, S Marwitz, B Baron-Lühr, G Zissel, C Kugler, K F Rabe, P Zabel, E Vollmer, J Gerdes, T Goldmann

**Affiliations:** 1 Clinical and Experimental Pathology, Research Center Borstel, Parkallee 1-40, D-23845 Borstel, Germany; 2 Tumor Biology, Research Center Borstel, Parkallee 1-40, D-23845 Borstel, Germany; 3 Department for Pneumology, University of Freiburg, Killianstrasse 5, D-79106 Freiburg, Germany; 4 Hospital Großhansdorf, Wöhrendamm 80, D-22927 Großhansdorf, Germany; 5 Medical Clinic, Research Center Borstel, Parkallee 1-40, D-23845 Borstel, Germany

**Keywords:** lung cancer, adenocarcinoma, marker, immunohistochemistry, TTF-1, SP-B

## Abstract

**Background:**

Different therapy regimens in non-small-cell lung cancer (NSCLC) are of rising clinical importance, and therefore a clear-cut subdifferentiation is mandatory. The common immunohistochemical markers available today are well applicable for subdifferentiation, but a fraction of indistinct cases still remains, demanding upgrades of the panel by new markers.

**Methods:**

We report here the generation and evaluation of a new monoclonal antibody carrying the MAdL designation, which was raised against primary isolated human alveolar epithelial cells type 2.

**Results:**

Upon screening, one clone (MAdL) was identified as a marker for alveolar epithelial cell type II, alveolar macrophages and adenocarcinomas of the lung. In a large-scale study, this antibody, with an optimised staining procedure for formalin-fixed tissues, was then evaluated together with the established markers thyroid transcription factor-1, surfactant protein-A, pro-surfactant protein-B and napsin A in a series of 362 lung cancer specimens. The MAdL displays a high specificity (>99%) for adenocarcinomas of the lung, together with a sensitivity of 76.5%, and is capable of delivering independent additional diagnostic information to the established markers.

**Conclusion:**

We conclude that MAdL is a new specific marker for adenocarcinomas of the lung, which helps to clarify subdifferentiation in a considerable portion of NSCLCs.

Lung cancer is one of the leading causes of death worldwide, with a still rising incidence (Van Lerberghe *et al*, 2008). There are long-standing differences between the treatment regimens for the main subtypes of lung cancer, which is primarily divided into small-cell lung cancer (SCLC) and non-SCLC (NSCLC). Differentiation among SCLC, NSCLC and also metastases is of large therapeutic relevance. With regard to this, appropriate immunohistochemical procedures have been developed (Kaufmann *et al*, 1997).

Novel chemotherapeutic approaches have recently been developed for NSCLC, which is largely known as a chemoresistant tumour. In the respective clinical studies, substantial differences between adenocarcinomas and squamous cell carcinomas or different types of large-cell carcinomas have been shown with regard to the adequate therapeutic regimens (Smit *et al*, 2001; Esteban *et al*, 2009; Kim *et al*, 2009; Lee *et al*, 2009). Therefore, subdifferentiation of NSCLC, which to some degree has been an academic issue in the past, is currently getting more into focus as increasingly becoming a check element within therapeutic decisions.

For such subdifferentiation strategies among NSCLC, different elements of the pulmonary surfactant system repeatedly proved to be reliable markers for adenocarcinomas of the lung. These comprise members of the surfactant proteins themselves, such as surfactant protein-A (SP-A) and pro-surfactant protein-B (SP-B), which are highly specific markers but lack sensitivity (Mizutani *et al*, 1988; Brasch *et al*, 2003). Thyroid transcription factor-1 (TTF-1), a positive regulator of surfactant protein promoter activity, has emerged as a major and sensitive marker for a lung cancer, but is not up to differentiate the different entities of pulmonary cancer, such as adenocarcinoma, large-cell neuroendocrine carcinoma, small-cell carcinoma or carcinoid (Folpe *et al*, 1999; Barlési *et al*, 2005). In addition, it designates thyroid carcinomas and their metastases (Boggaram *et al*, 2003; Kargi *et al*, 2007). The aspartic protease family member napsin A is involved in processing of SP-B in alveolar epithelial cell type II (AECII), and can be used as a marker for adenocarcinomas of the lung (Chuman *et al*, 1999; Hirano *et al*, 2003; Dejmek *et al*, 2007).

Despite the information obtained by the use of these established markers, there is still a need for additional diagnostic information in a portion of NSCLC.

With regard to this, we initialised a screening approach starting with immunisation of mice against human primary AECII (Zissel *et al*, 2000). After generation of numerous hybridomas, the corresponding monoclonal antibodies were subjected to primary screening using cell cultures and human tissues. One of the clones directed against a cytoplasmic fraction of AECII showed reactivity with AECII, alveolar macrophages and adenocarcinomas of the lungs, which was further verified using tissue microarrays (TMAs) from NSCLC tissues treated with the HOPE technique (Srinivasan *et al*, 2002; Goldmann *et al*, 2003, 2005). This clone was designated MAdL (marker for adenocarcinomas of the lung). Subsequently, an optimised protocol for the use of MAdL on formalin-fixed, paraffin-embedded (FFPE) tissues was developed. Here, we present the results of the use of MAdL in addition to the above-mentioned established markers.

## Materials and methods

### Primary human AECII

Samples from macroscopically tumour-free lung tissue were cut from the surgical specimens and used for cell isolation procedure as described previously (Pechkovsky *et al*, 2005, 2006). In brief, the lung tissue was first sliced and slices were washed three times at 4 °C in PBS. The washed slices were incubated in sterile dispase solution at 37 °C for 45 min. After dispase digestion, the lung tissue slices were cut into small, pipetable pieces and thoroughly pipetted for several minutes. Crude tissue and cell suspensions were filtered through nylon gauze with meshes of 100 *μ*m, 50 *μ*m and 20 *μ*m. The resulting single-cell suspension was placed on Ficoll separating solution and centrifuged at 800 **g** for 20 min. The AECII-enriched cells from the interphase were washed and incubated in 100 mm plastic dishes at 37 °C in humidified air containing 5% CO_2_ for 15, 20 and 30 min, with seeding of non-adherent cells on fresh dishes for each time interval to remove adherent cells (alveolar macrophages, monocytes, fibroblasts and endothelial cells). To remove remaining monocytes/macrophages and lymphocytes, antibodies against CD3 (OKT3 and ECACC 86022706) and CD14 (HB-246 ATCC) were added and the antibody-binding cells were removed by anti-mouse IgG-coated magnetic beads and magnetic activated cell sorting (MACS) system (Miltenyi Biotec, Bergisch Gladbach, Germany), as suggested by the supplier. Identity of type II alveolar epithelial cells was confirmed by a modified Papanicolaou staining, their alkaline phosphatase activity and SP-A mRNA expression in RT-PCR (see below). Cell purity was assessed by immunoperoxidase staining with monoclonal antibodies directed against CD3 and CD14 (Immunotech, Marseille, France) as previously described (20). Viability of the AECII after isolation was >97%, as determined by trypan blue exclusion. After the final step of MACS purification, the AECII preparations included in this report were free of CD14+ and CD3+ cells, as determined by immunocytochemistry. In all, 98±1.3% of cells were identified as AECII by the presence of dark blue inclusions, as revealed by modified Papanicolaou staining, and 93±2.1% of cells were positive for alkaline phosphatase (data not shown).

### Generation of the monoclonal antibody

A total of 1 × 10^7^ cells were thawed and washed twice in 0.9%. NaCl solution. Subsequently, the cells were lysed in 1 ml lysis solution (0.9% NaCl and 0.5% Tween 20) and vortexed for 1 min. The lysate was cleared by centrifugation at 34 **g** for 15 min and the supernatant stored at −20 °C. A female Balb C mouse was immunised by subcutaneous injections using a mixture of cell lysate and GERBU Adjuvant MM (Gerbu Biotechnik, Gaiberg, Germany): day 0, 60/40 *μ*l; days 14 and 21, 30/20 *μ*l; and days 28, 29 and 30, 50 *μ*l lysate only. To generate antibody-producing hybridoma lines, spleen cells of this mouse were fused on day 31 with × 63 Ag8.6.5.3 myeloma cells using a standard PEG-based procedure (Köhler and Milstein, 1975). Antibody clones were assessed for their staining pattern using cytospins of primary AECII cells as well as sections of normal human lung and tonsil.

### Immunohistochemistry

For a preliminary study of the immune reactivity of MAdL, TMAs were produced from 35 HOPE-fixed, paraffin-embedded NSCLC tissues as previously described (Goldmann *et al*, 2005). HOPE-fixed specimens allow immunohistochemistry (IHC) without antigen retrieval (AR), which could create artefacts in staining or morphology (Goldmann *et al*, 2003).

For immunodetection of MAdL, 1 *μ*m thick sections of HOPE-fixed, paraffin-embedded tissues were deparaffinised by two times of incubation in isopropanol for 10 min at 65 °C. Deparaffinised sample slides were air dried at room temperature and rehydrated for 10 min in 70% (v/v) acetone/DEPC-treated water at 4 °C. Remaining acetone was removed by incubation for 10 min in DEPC-treated water at 4 °C and transferred into distilled water at room temperature. Endogenous peroxidases were blocked for 10 min in 3% H_2_O_2_ solution. A volume of 2 *μ*g ml^−1^ of isolated MAdL was diluted with antibody diluent (Zytomed Systems, Berlin, Germany) and applied for 60 min in a moist chamber. For blocking and detection, a HRP-conjugated polymer kit according to the manufacturer's instructions (Zytomed Systems) was used. Washing steps were carried out three times for 5 min after each reagent step with washing buffer (50 mM Tris saline buffer with 0.1% (v/v) Tween 20; pH 7.6). Negative controls were included under omission of secondary antibody. Permanent AEC (permanent AEC Kit, Zytomed Systems) was used as substrate for HRP-conjugated polymer. Colour reaction was stopped with distilled water. Samples were dehydrated in increasing concentrations of ethanol, washed for 20 s in xylene and cover slips were mounted using Pertex (Medite, Burgdorf, Germany) as mounting medium.

### IHC with MAdL on FFPE tissues

As most tissue specimens are classically fixed with 4% neutral-buffered formalin in routine diagnostics, IHC has usually faced the cross-linking effects (Srinivasan *et al*, 2002) before conducting immunodetection. To increase the diagnostic value, MAdL had therefore to be applicable on FFPE specimens. For an optimal staining result on FFPE specimens, heat-induced AR was tested with acidic, as well as alkaline buffers and standard enzymatic digests (see Table 1). Finally, enzyme-based pretreatment with Fast Enzyme (Zytomed Systems) for 3 min at ambient temperature or Proteinase XXV (Thermo Fisher Scientific, Waltham, MA, USA) for 10 min at ambient temperature turned out to provide the best results.

Immunohistochemistry with FFPE tissues was generally conducted as mentioned above, with the exception that FFPE-tissue slides were deparaffinised by incubation in xylene (2 × 10 min) and subsequent rehydration in a graded ethanol series for 2 min each step (2 × 100, 2 × 96, 90, 80, 70%, 2 × distilled water). The primary antibodies were applied for 1 h at ambient temperature as described in Table 2. Negative controls were included under omission of primary antibody in each staining series, as well as positive reference sections from human lung to ensure even results.

### Screening for cross-reactivity in malignant and non-malignant human tissues

For evaluation of affinity and cross-reactivity in human tissues, the antibody was tested on various malignant pulmonary (see Table 3) and non-malignant tissues (see Table 4) from either the lung or the other functional systems. In short, the expression of MAdL, in addition to its expression in human lung adenocarcinomas, was investigated in the most common lung-metastasising tumours. To exclude false-positive reactions with other non-malignant tissues, MAdL was analysed for expression in any functional system, including digestive and urogenital tract, as well as connective tissue, nervous system, endocrine organs and the skin.

### Study group

Patient materials were obtained from either lobectomy, pneumonectomy or peribronchial biopsy at the Hospital of Großhansdorf or Medical Clinic Borstel, Germany. All used archived FFPE tissue blocks were of pathologically and clinically proven diagnosis.

The expression of MAdL was evaluated and compared with common markers for adenocarcinomas of the lung in 362 primary lung carcinomas. The group consisted of 154 squamous cell carcinomas, 167 adenocarcinomas, 2 adenosquamous carcinomas, 19 small-cell carcinomas, 17 large-cell carcinomas and 3 carcinoids. From each diagnosis, an almost comparable amount of specimens was used from either surgical or biopsy origin, in order to compare possible expression differences (260 surgical specimens and 201 biopsy specimens). In addition to primary lung carcinomas, expression of MAdL was investigated in 111 non-pulmonary carcinomas. This series comprised of 28 colon carcinomas, 19 mamma carcinomas, 11 prostate carcinomas, 6 pancreas carcinomas, 10 gastric carcinomas, 21 kidney carcinomas, 1 bile duct carcinoma, 1 hepatocellular carcinoma, 1 thyroid carcinoma, 3 endometrium carcinomas and 2 urothelium carcinomas, as well as 8 cases of epitheloid mesothelioma. Detailed diagnostic information for each diagnosis is summarised in Table 3.

Diagnosis and grading of investigated specimens was conducted according to the WHO Classification of Tumours 2004.

## Results

### Establishing of IHC and screening of non-malignant tissues

In a prescreening study, HOPE-fixed carcinomas of the lung were investigated by application of MAdL culture supernatants without any AR. In all, 80% of investigated adenocarcinomas (16 out of 20) were positive and displayed a cytoplasmic, granular signal in epithelia of the tumour. All tested squamous cell carcinomas (20) were negative for MAdL (data not shown).

Results of the prescreening study were verified on formalin-fixed adenocarcinomas under equal conditions after establishing optimal AR conditions. A broad range of commonly used AR methods has been applied and compared to optimise the MAdL staining protocol with FFPE tissues. No heat-induced AR, with both acidic and alkaline buffer, resulted in reasonable staining. The same holds true for enzymatic digests with proteinase K, trypsin and pepsin. The best results were obtained by applying fast enzyme and proteinase XXV. Saponin was used as a permeabilisation agent (Pignal *et al*, 1982) and Ficin provided positive staining, but the staining was less intense when compared with fast enzyme or proteinase XXV treatment (Table 1).

**Figure 1:**
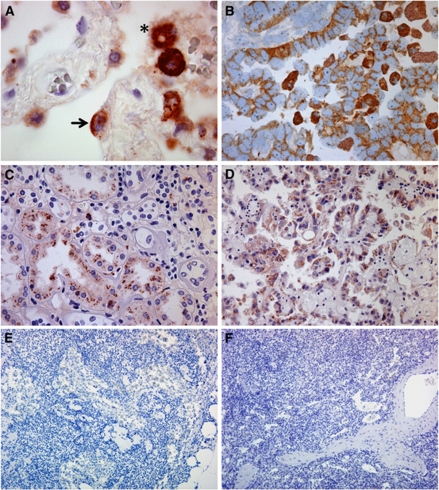
Photomicrograph of IHC on FFPE tissues targeting MAdL, with red colour indicating positive signal. Immunoreactivity of MAdL in AECII (indicated with arrow) and intra-alveolar macrophages (indicated with star; **A**, × 1000, indicated with arrows), as well as a case of corresponding adenocarcinoma of the lung (**B**, × 400) is depicted in the upper part. Non-malignant tissues of proximal kidney tubules (**C**, × 200) as well as chromophile renal cell carcinoma (**D**, × 200) are shown in the centre lane. Lymph node (**E**, × 200) and spleen tissue (**F**, × 200) housing non-alveolar macrophages are all negative for MAdL.

After the setup of a standard staining protocol for FFPE tissues, immunolocalisation of MAdL displayed positive cytoplasmic signals in AECII as well as in intra-alveolar macrophages (Figure 1A). No signal was seen in bronchi and referring glands, respiratory epithelia, type 1 pneumocytes, mesenchymal cells and inflammatory infiltrates. To exclude any false-positive signals, a variety of non-malignant and non-respiratory tissues were further investigated for expression of MAdL. Within the analysed tissues, only the proximal tubules of the kidney displayed immunoreactivity with MAdL (Figure 1C and Table 4), this is in line with its expression in chromophile renal cell carcinoma (Figure 1D). No staining was found in lymphoid tissue (Figure 1E and F).

### Comparison of MAdL expression with common markers for adenocarcinomas of the lung

For investigation of MAdL expression and comparison with common applied markers for diagnosis of adenocarcinomas of the lung, a cohort of lung carcinomas was screened. All specimens were analysed for expression of MAdL, TTF-1, SP-A, SP-B and, in case of squamous cell carcinoma, cytokeratin 5/6.

**Figure 2:**
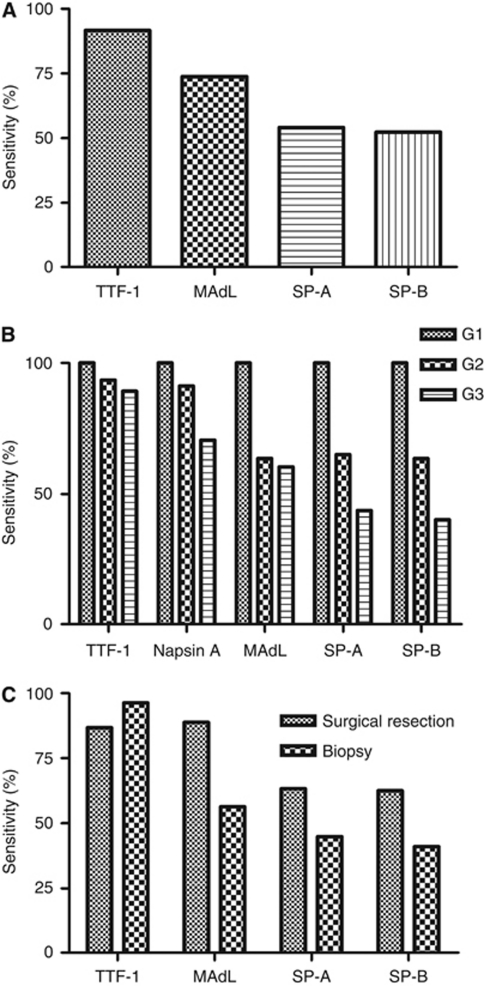
Bar plot depicting general sensitivity (**A**) of applied IHC markers on 167 adenocarcinomas of the lung and sensitivity depending on grading (**B**) or specimen origin (**C**). Thyroid transcription factor-1 was expressed in 154 out of 167 (92.2%) cases of adenocarcinomas and MAdL in 124 out of 167 (74.2) cases. The surfactant proteins SP-A and SP-B were found to be expressed in 92 (55%) and 88 (52.6%) of 167 samples, respectively.

Results for all the markers are displayed in Table 3, including histomorphological entities and expression profiles. In case of adenocarcinomas, 90.2% showed positive expression of TTF-1 and 74.2% were positive for MAdL. Staining targeting the surfactant proteins SP-A and SP-B revealed 55% and 52.6% positivity, respectively (Figure 2A). No expression of TTF-1, MAdL, SP-A and SP-B could be observed in squamous cell carcinomas, whereas all of them displayed a positive signal for cytokeratin 5/6. Neither small-cell and large-cell carcinomas, nor carcinoids were positive for MAdL or surfactant proteins. On the contrary, TTF-1 expression was observed in 73.6% of small-cell carcinomas, in 23.5% of large-cell carcinomas and in 66.6% of atypical carcinoids. The two investigated adenosquamous lung tumours were all positive for TTF-1 and MAdL in adenoid-differentiated part and CK 5/6 in squamous-differentiated part, respectively. Only one case was positive for both SP-A and SP-B. Within the group of non-pulmonary tumours, MAdL immunoreactivity could only be observed in one case of chromophile renal cell carcinoma (Figure 1D). Other investigated subtypes of renal carcinoma such as papillary and clear-cell renal cell carcinoma showed no expression.

### Marker expression depending on tumour grading and specimen origin

Regarding the grading of investigated adenocarcinomas, only a small number (four) were low-grade (G1) carcinomas. The majority (77 cases) consisted of intermediate- (G2) and 87 cases of high-grade carcinomas (G3). In routine diagnostics, the intermediate- and high-grade adenocarcinomas of the lung are far more frequent. Therefore, it is helpful to have a marker at hand, which maintains its expression qualitatively even in poorly differentiated carcinomas. With respect to TTF-1, its expression did not reduce dramatically in intermediate- and high-grade adenocarcinomas. The novel aspartic proteinase napsin A showed a comparable sensitivity in intermediate carcinomas to TTF-1. In high-grade carcinomas, its expression declined to a sensitivity of 70.6%. The MAdL revealed the same sensitivity as the other markers in low-grade adenocarcinomas. There is no notable decline in sensitivity from intermediate- to high-grade cases, albeit the overall sensitivity is less than TTF-1 and napsin A. Surfactant protein-A and -B share a comparable sensitivity with MAdL in intermediate-grade cases, whereas their expression ceased notably in high-grade carcinomas (Figure 2B).

As a growing number of samples results from diagnostic biopsies, we intended to analyse and compare the sensitivity of MAdL and other markers between specimens of surgical and biopsy origin. Regarding TTF-1, there was a high sensitivity (>75%) in both surgical and biopsy specimens. Interestingly, it could be observed that in surgical specimens, the sensitivity of MAdL was even a little bit higher compared with TTF-1. The surfactant proteins SP-A and SP-B showed a moderate expression in surgical tissues (57% and 56%, respectively). Emphasising on biopsy tissues, MAdL displayed moderate sensitivity (56%) in contrast to SP-A and SP-B, which declined to 44.9 and 41%, respectively (Figure 2C).

### Expression patterns of TTF-1, MAdL, SP-A and SP-B in adenocarcinomas of the lung

**Figure 3:**
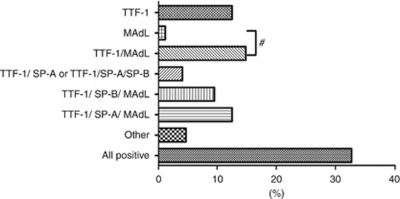
Observed expression patterns of applied IHC markers for 167 cases of adenocarcinomas of the lung. Within the observed patterns, 55 (32.7%) cases were positive for all the markers. Thyroid transcription factor-1 alone was observed in 21 cases (12.5%) and MAdL alone in 2 cases (1.1%). Thyroid transcription factor-1 and MAdL as the only expressed markers were observed in 25 cases (14.9%), whereas TTF-1/MAdL/SP-A or TTF-1/MAdL/SP-B stated for 21 (12.5%) or 16 (9.5%) cases, respectively. Expression of TTF-1 and SP-A or TTF-1 with both surfactant proteins counted for each seven (4.1%) cases. Less frequent combinations of markers included TTF-1/SP-B, MAdL/SP-A, MAdL/SP-B, MAdL/SP-A/SP-B and SP-A/SP-B and were grouped as ‘other’ with 4.7%. No expression of any marker was observed in six cases (3.5%). The possible diagnostic benefit of MAdL is displayed with ‘#’ and accounts for 27 cases (16%) within the investigated collective.

Among the investigated 167 cases of adenocarcinomas, the majority was positive for all the applied markers (32.7%). Thyroid transcription factor-1 expression alone was observed in 21 (12.5%) and MAdL alone in 2 (1.2%) cases. Within the investigated group, the expression of both TTF-1 and MAdL comprised about 25 cases (14.9%) and was the second most prevalent observed pattern. A combination of either TTF-1, MAdL and SP-B (9.5%), or TTF-1, MAdL and SP-A (12.5%) was of third most incidence (Figure 3).

### MAdL in comparison with TTF-1, SP-A and SP-B

**Figure 4:**
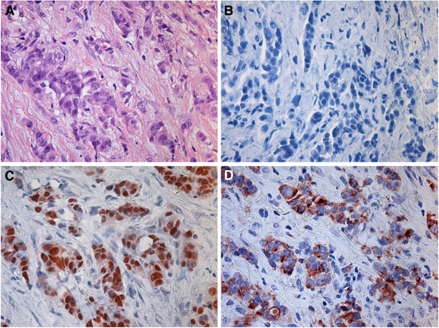
Photomicrograph of IHC on a case of pleura carcinosis from pulmonary adenocarcinoma origin. Hematoxylin–eosin-stained overview (**A**). No expression of either SP-A or SP-B was observed (data only shown for SP-A; **B**). Targeting TTF-1 resulted in strong nuclear (**C**) or cytoplasmic staining for MAdL (**D**). All images were at × 400 magnification.

**Figure 5:**
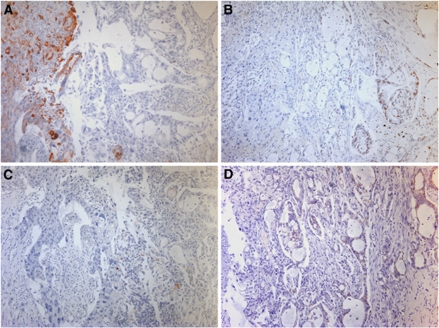
Photomicrograph of IHC on a case of adenosquamous carcinoma of the lung. Squamous-differentiated tumour component revealed a strong CK5/6 positivity (**A**). Adenoid component of the tumour shows a distinct nuclear signal for TTF-1 (**B**) and a patchy staining for SP-B (**C**). Cytoplasmic signals for MAdL could be observed, in contrast to TTF-1, in the majority of glands (**D**).

In addition to routine cases in diagnostics, there are repeating situations with aberrant histological specimen. As depicted in Figure 4, a pleura carcinosis from a pulmonary adenocarcinoma was negative for both SP-A and SP-B. Addressing TTF-1 could only confirm pulmonary origin of the carcinoma, although analysis of MAdL expression revealed the specific subtype. The same holds true for mixed tumour entities, which might have a squamous- as well as an adenoid-differentiated component (Figure 5). Cytokeratins 5 and 6 detect the squamous component in the left part of the photomicrograph, and TTF-1 is found to be expressed in a subgroup of glands. In contrast to SP-B, which only provides a patchy and weak signal, MAdL is expressed in the majority of adenoid glands.

## Discussion

### Establishing MAdL staining protocol

The requirements of antibodies as markers in daily routine diagnostics have to face the molecular characteristics of the specimen analysed. As most tissues are traditionally FFPE, an AR is indispensable because of formalin's cross-linking drawbacks (Srinivasan *et al*, 2002). New markers have to meet these circumstances in order to become widely applicable. As it is hypothesised that most adenocarcinomas probably arise from bronchoalveolar stem cells, from which Clara cells or AECII derive (Kim *et al*, 2009), we immunised mice with a cytoplasmic fraction of AECII to generate a diagnostically relevant antibody to differentiate adenocarcinomas of the lung. One of the clones, designated MAdL, demonstrated a high specificity for pulmonary adenocarcinomas in HOPE-fixed tissues. Additionally, MAdL reacts with AECII and alveolar macrophages, whereas macrophages from other locations showed no reaction (Figure 1E and F). This may suggest phagocytosis rather than synthesis of the MAdL antigen in macrophages. To expand the application spectrum to FFPE tissues, an extensive array of pretreatments was investigated for an optimal staining result. Here, we present a newly generated, applied and evaluated antibody for differentiation of adenocarcinomas from squamous cell carcinomas of the lung, in addition to the optimal application protocol.

### Diagnosis of lung cancer

Lung cancer is the most common and deadliest cancer in the world. Among the 12.7 millions of cancer incidence worldwide in 2008, lung cancer accounts for 13% or more than 1.4 million cases with fatal outcome (Jemal *et al*, 2011). Within the last decades, adenocarcinoma has become the most prevalent subtype of lung cancer (Boyle and Levin, 2009), which accounts for almost half of diagnosed cases (Curado *et al*, 2007). Over a long period, the simple discrimination between SCLC and NSCLCs used to be sufficient regarding therapeutic intervention. As new treatments such as growth factor receptor mutation analyses offer targeted therapies, the precise differentiation of NSCLC has to keep up with these developments (Ramalingam *et al*, 2011). As diagnostics usually have to cope with scarce tumour material, which hardly allows satisfactory differentiation of tumour entities by morphology and heterogeneity of the material is well known, development and application of specific markers are crucial. The majority of metastases within the lung are adenocarcinomas (Dail and Hammar, 2008). Therefore, discrimination between primary lung adenocarcinoma and metastasis influences the subsequent therapies (Tanaka *et al*, 2008; Zheng and Fernando, 2010). Routine diagnostic procedures are based on morphology and immunohistochemical detection of specific antigens. Up to date, there is still little consensus about the applied antibodies or other standards. The antibody panel is highly heterogenous, and depending on the study it includes up to five different markers (Ring *et al*, 2009).

### MAdL delivers high sensitivity and specificity for adenocarcinomas of the lung

Thyroid transcription factor-1 is commonly used as a basic marker for lung carcinomas, with a reported sensitivity range for lung adenocarcinomas between 75 and 80% (Lau *et al*, 2002; Jagirdar, 2008) and 92% according to our own study (see Table 3): However, TTF-1 can also be found in other lung tumour entities such as SCLC, large-cell neuroendocrine carcinomas and carcinoids (Folpe *et al*, 1999). Additionally, TTF-1 is reported to be expressed inversely in relation to tumour differentiation (Lau *et al*, 2002; Jagirdar, 2008; Yang and Nonaka, 2010). Regarding the reactivity with other (Klingen *et al*, 2010) and lung malignancies, TTF-1 is not sufficient as a stand-alone marker for diagnosis of primary lung adenocarcinomas. In addition, SP-B and SP-A are usually applied to further discriminate between squamous cell carcinomas and adenocarcinomas. For SP-A, the sensitivity ranges between 45 and 64%, and its expression declines notably with loss of differentiation (Yang and Nonaka, 2010). As the highly specific SP-A clone PE-10 (Dempo *et al*, 1987; Sugiyama *et al*, 1992; Goldmann *et al*, 2009) is no more commercially available and the follow-up clone displays cross-reactivity with intestinal epithelia and carcinomas (authors own observations), the specificity for pulmonary adenocarcinomas is not warranted. Therefore, for means of differentiation, SP-B can be applied but with a lack of sensitivity (52.6%, see Figure 2A). Applying MAdL as a second-line marker improves sensitivity to 74.2% in contrast to the commonly used surfactant proteins. Hence, we were able to show in an extensive study that MAdL is only expressed in alveolar macrophages, AECII and proximal tubules of the kidney in non-malignant tissues. Emphasising on malignant tissues, MAdL can exclusively be found in adenocarcinomas, whereas adenoid-differentiated extrapulmonary malignancies, which usually metastasise to the lung (Dail and Hammar, 2008), were not detected. Furthermore, only adenoid parts of adenosquamous carcinomas, as well as one case of chromophile renal cell carcinoma, displayed positivity for MAdL. This reactivity may come in line with the observed expression in its non-malignant tissue origin, the proximal renal tubules (Figure 1A). In contrast to TTF-1, no reactivity with non-adenous lung carcinomas, such as neuroendocrine carcinomas, large-cell neuroendocrine carcinomas, as well as small-cell bronchial carcinoma and carcinoids, was observed. This counts for a superior quality of MAdL as a second-line marker for subdifferentiation of lung cancer. Owing to the way of generating MAdL by immunisation of mice with preparations of fractions of primary human AECII, we do not yet know the target molecule of MAdL. The knowledge of this target molecule would allow insights into the biological relevance of MAdL expression in different tissues. Appropriate studies are currently underway to uncover this target molecule, which use techniques such as two-dimensional gel electrophoresis, immunoprecipitation techniques, accompanied by mass spectrometry and immunogold electron microscopy.

### Sensitivity of MAdL persists in biopsy material and during dedifferentiation

Biopsy specimens, in general, inhabit only a small fraction of the whole tumour compared with surgical specimens. Therefore, the heterogeneity in expression of applied markers has a more severe effect on the sensitivity. In contrast to SP-A (44.9%) and SP-B (41%), MAdL retained a higher sensitivity (56.4%) in biopsy material. Interestingly, MAdL (88.8%) showed a comparable sensitivity to TTF-1 (86.7%) in surgically resected specimens (Figure 2C). In addition to sensitivity in biopsy specimen, we further investigated the sensitivity of MAdL depending on tumour grading. In general, there is up to date no universal grading system for adenocarcinomas of the lung that is not disputed (Travis *et al*, 2011). However, it is widely accepted that loss of differentiation by increasing grading reflects likelihood of lymph node metastasis and reoccurrence after surgical intervention (Chung *et al*, 1982). Thyroid transcription factor-1 and napsin A both show an impressing sensitivity almost independent of grading, but with a noteworthy lack of specificity for adenocarcinomas of the lung. The surfactant proteins range lower in sensitivity and suffer, in addition, from a loss in G2 to G3 tumours. The MAdL as a marker with sensitivity in between napsin A and SP-A or SP-B will add a decent advantage based on its specificity and persistent expression in terms of grading stages or specimen origin.

### Diagnostic benefit of applying MAdL

The currently arising targeted therapies comprising receptor tyrosine kinase inhibitors such as gefitinib or erlotinib offer a true benefit for the patients (Mok *et al*, 2009). With regard to the therapy decision, one has to bear in mind that this is not exclusively achieved by molecular-based analysis as for EGFR mutations (Rosell *et al*, 2009), but also deeply depends upon the previous diagnosis of NSCLC and subdifferentiation into adenocarcinomas. Without a concrete and specific diagnosis, the subsequent molecular-based analyses and therapies will be impaired. Here, MAdL as a new and specific marker for adenocarcinomas of the lung offers a diagnostic benefit of 16% if used as a second-line marker besides TTF-1. As 12.7 × 10^6^ cases of cancer have been globally diagnosed in 2008, with 13% (1.6 × 10^6^) of which are lung cancers (Jemal *et al*, 2011), with almost 50% of adenocarcinomas (Curado *et al*, 2007), a 16% higher diagnosis because of MAdL would account for 132 000 more cases each year worldwide. In addition to EGFR-targeting therapies, new molecular insights into lung cancer development currently have emerged. K-RAS mutations appear in 25% of adenocarcinomas and may be a promising target of new therapeutic strategies (Sunaga *et al*, 2011), as well as the recently described EML4 protein (Radtke *et al*, 2010) and its EML4-ALK oncoprotein counterpart (Gerber and Minna, 2010). This growing number of insights into molecular events during lung cancer development will doubtlessly lead to better therapy, but the diagnosis of certain NSCLC subtypes has to keep pace with these events. Therefore, we developed MAdL as a new second-line marker for adenocarcinomas of the lung that might improve the primary diagnoses on which targeted therapies depend.

## Figures and Tables

**Table 1:** Assessment of AR for MAdL on FFPE specimens

**Result (+/−)**	**Tested AR**	**Applied conditions**	**Producer**
+++	Fast enzyme	3 min ambient temperature	Zytomed Systems, Berlin, Germany
++	Proteinase XXV	10 min ambient temperature	Thermo Fisher Scientific, Waltham, MA, USA
+	Ficin	5–10 min, 37 °C	Zytomed Systems, Berlin, Germany
			
−	Saponin (0.05%)	30 min ambient temperature	Serva Electrophoresis, Heidelberg, Germany
−	Proteinase K	10 min, 37 °C	Qiagen, Hilden, Germany
−	Pepsin	10 min, 37 °C	Merck, Darmstadt, Germany
−	Citric acid buffer, pH 6	30 min, 90 °C	Self-mixed (10 mM citric acid, 0.05% Tween 20)
−	Tris-EDTA, pH 9	30 min, 90 °C	Zytomed Systems, Berlin, Germany

Abbreviations: AR=antigen retrieval; FFPE=formalin fixed, paraffin embedded. Result: −=unsufficient staining quality; +=week staining quality; ++=moderate staining quality; +++=good staining quality.

**Table 2:** Applied antibodies and AR for immunohistochemistry

**Antigen**	**Clone and producer**	**Dilution (*μ*g ml^−1)^**	**Applied antigen retrieval**
MAdL	MAdL, Research Center Borstel, Germany	2	Fast enzyme, 3 min at ambient temperature
TTF-1	SPT24, DCS, Hamburg, Germany	1/300	Citric acid buffer, pH 6, 30 min, 90 °C
SP-A	PE-10, DCS, Hamburg, Germany	1/200	Tris-EDTA, pH 9, 30 min, 90 °C
SP-B	SPM-158, Zytomed Systems, Berlin, Germany	1/50	Citric acid buffer, pH 6, 30 min, 90 °C
Napsin	KCG1.1, Zytomed Systems, Berlin, Germany	1/200	Citric acid buffer, pH 6, 30 min, 90 °C
CK 5/6	D5/16B4, DakoCytomation, Glostrup, Denmark	1/100	Tris-EDTA, pH 9, 30 min, 90 °C

Abbreviations: AR=antigen retrieval; CK=cytokeratin; SP=surfactant protein; TTF=thyroid transcription factor.

**Table 3:** Investigated specimens

			**Specimen origin**		**Expression of marker molecules *N* (%)**				
	**Tumour entity (*N*, sex ratio, m/f, mean age)**	**Grading (*N*)**	**Surgical**	**Biopsy**	**TTF-1**	**MAdL**	**SP-A**	**SP-B**	**CK 5/6**
**Primary lung carcinomas**	Squamous cell carcinoma (154, 122/32, 66.7)	G1 (9), G2 (63), G3 (82)	77	77	0	0	0	0	154 (100)
	Adenocarcinoma (167, 84/83, 63)	G1 (4), G2 (77), G3 (87)	87	78	154 (92.2)	124 (74.2)	92 (55)	88 (52.6)	0
	Small-cell carcinoma (19, 10/9, 66.3)	G3 (19)	4	5	14 (73.6)	0	0	0	0
	Large-cell carcinoma (17, 16/1, 62.76)	G3 (17)	4	13	4 (23.5)	0	0	0	0
	Adenosquamous carcinoma (2, 1/1, 61.5)	G2 (2)	2	0	2 (100)	2 (100)	1 (50)	1 (50)	2 (100)
	Carcinoids (3, 1/2, 68.7)	G2 (3)	0	3	2 (66.6)	0	0	0	0
									
**Other carcinomas**	Colon carcinoma (28, 7/11, 72.3)	G2 (18), G3 (10)	24	4		0			
	Mamma carcinoma (19, 0/19, 60.5)	G1 (1), G2 (7), G3 (11)	14	5		0			
	Mesothelioma (8, 7/1, 69.7)	G2 (1), G3 (7)	0	8		0			
	Prostate carcinoma (11, 11/0, 64)	G2 (5), G3 (6)	11	0		0			
	Pancreas carcinoma (6, 3/3, 63.6)	G2 (1), G3 (5)	6	0		0			
	Gastric carcinoma (10, 9/1, 78.6)	G2 (4), G3 (6)	4	6		0^a^			
	Renal carcinoma (21, 11/10, 69)	G1 (2), G2 (9), G3 (10)	21	0		1 (4.7)^b^			
	Bile duct carcinoma (1, 1/0, 79)	G3 (1)	0	1		0			
	Hepatocellular carcinoma (1, 1/0, 45)	G2 (1)	1	0		0			
	Endometrium carcinoma (3, 0/3, 68)	G2 (2), G3 (1)	3	0		0			
	Thyroid carcinoma (1, 0/1, 64)	G2 (1)	1	0		0			
	Urothelium carcinoma (2, 2/0, 70)	G2 (1), G3 (1)	1	1		0			

Abbreviations: CK=cytokeratin; f=female; m=male; SP=surfactant protein; TTF=thyroid transcription factor.

a Not relevant for diagnostics.

b Chromophile renal cell carcinoma.

**Table 4:** Expression of MAdL in non-malignant tissues

**Investigated tissues**	**No expression**	**Expression**
Respiratory system	Respiratory epithelia	Pneumocyte type II
	Peribronchial glands	Alveolar macrophages
	Pneumocyte type I	
		
Digestive tract	Gastric mucosa	
	Duodenum mucosa	
	Small-intestine mucosa	
	Colon mucosa	
	Liver parenchyma	
	Bile duct and bladder	
	Pancreas parenchyma	
		
*Urogenital tract*		
Kidney	Tubules, glomeruli	Proximal tubules
Efferent urinary system	Urothelia	
Prostate	Seminal vesicle	
Testis	Seminal epithelia	
		
Connective tissue	Smooth/skeletal muscles	
	Heart muscle	
	Adipocytes	
	Fibroblasts	
		
Nervous system	Nerve (autonomous and somatic)	
	Ganglions	
	Brain	
		
Endocrine organs	Adrenal gland	
	Pituitary gland	
	Thyroid gland	
	Parathyroid gland	
	Langerhans islet cells	
		
Skin	Epidermis	
	Melanocytes	
	Integumentary appendage	
